# Epitaxial Growth and Characterization of 4H-SiC for Neutron Detection Applications

**DOI:** 10.3390/ma14040976

**Published:** 2021-02-19

**Authors:** Alessandro Meli, Annamaria Muoio, Antonio Trotta, Laura Meda, Miriam Parisi, Francesco La Via

**Affiliations:** 1 CNR-IMM, Headquarter, Strada VIII 5, 95121 Catania, Italy; annamaria.muoio@imm.cnr.it; 2 Dipartimento di Fisica e Astronomia Ettore Majorana, Università degli Studi di Catania, Via S. Sofia 64, 95123 Catania, Italy; 3 ENI-MAFE, Via A. Pacinotti 4, 30175 Venezia, Italy; antonio.trotta@eni.com (A.T.); miriam.parisi@eni.com (M.P.); 4 ENI—Renewable Energy and Environmental R&D Center, Via G. Fauser, 4, 28100 Novara, Italy; laura.meda@eni.com

**Keywords:** 4H-SiC, neutron detector, epitaxial growth, micro-Raman, i-LOPC

## Abstract

The purpose of this work is to study the 4H-SiC epitaxial layer properties for the fabrication of a device for neutron detection as an alternative material to diamond detectors used in this field. We have studied a high growth rate process to grow a thick epitaxial layer (250 µm) of 4H-SiC and, in order to estimate the quality of the epitaxial layer, an optical characterization was done through Photoluminescence (PL) spectroscopy for stacking fault defect evaluation. Micro Raman spectroscopy was used for simultaneous determination of both carrier lifetime and induced carriers in equilibrium. We have compared these results with other two samples with an epitaxial layer of 100 micron, obtained with two different growth rates, 60 and 90 µm/h, respectively. From Raman measurements it has been observed that both the growth rate and the grown epitaxial layer thickness have an effect on the measured carrier lifetime. A comparison between different kinds of stacking faults (SF) was done, evaluating the influence of these defects on the carrier lifetime as a function of the injection level and it was observed that only at a low injection is the effect on the carrier lifetime low.

## 1. Introduction

Silicon Carbide (SiC) is a semiconductor used in a wide variety of application fields. Thanks to its properties, in particular its radiation hardness, it can be used in harsh environments, such as high temperatures and an ionizing radiation ambient. The high hexagonality rate value determines a wide bandgap, implying a high refractive index and a transparency to visible light. SiC shows a great variety of crystalline structures defined as crystalline polytypes, due to the sequence of the stacking of its atoms in a crystal lattice. Among these, the SiC polytype, 4H-SiC, shows the highest hexagonality rate, and therefore a higher bandgap and a higher isotropic mobility than other SiC polytypes used in microelectronics [1].

SiC detectors are more resistant in radiation environments than silicon detectors [2,3], therefore they can be used in environments where silicon detectors would fail, such as for neutron detection in nuclear fusion reactors. Studies have highlighted the high radiation hardness of SiC devices by observing the effects of neutron, proton and heavy ion irradiation on these diodes [4]. Therefore, for all these characteristics the use of these detectors is particularly interesting for all those activities where high particles flux must be detected, such as fast neutrons detection [5] in thermonuclear fusion.

A chemical vapor deposition (CVD) process epitaxially grows the high quality material used in device application. Epitaxy allows a highly precise control of doping, homogeneity and thickness of crystal films. Horizontal hot-wall reactors are typically used for the epitaxial growth, in a low pressure regime and temperature, for 4H-SiC, ranging from 1550 to 1650 °C [6]. Nowadays, 4H-SiC can be grown in a relatively large area (150 mm wafers) with thickness of about 250 µm or 280 µm and a low density of defects [7], and also free-standing, which is managed by electrochemical etching of the substrate [8].

Some growth parameters, such as the growth rate condition and Si/C ratio, have a great influence on the defect formation or annihilation [9]. For example, most single Shockey faults (SSFs) result from basal plane dislocations (BPDs) of the substrate, so a reduction of BPDs also produces a low density of single Shockley faults (SSFs), as reported by Kimoto and La Via [10,11]. SSFs lead to a decline in device performance by hindering the commercialization of stable, efficient and competitive bipolar devices. It has been observed that SFs induce a considerable increase of the recombination current and that this recombination is strongly dependent on the level introduced in the band-gap of the particular stacking faults [12].

**Figure 1:**
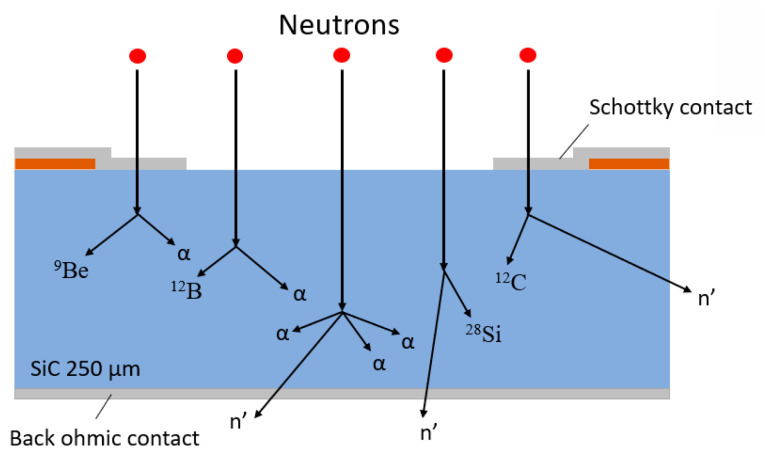
Scheme of the common interactions that take place on the epitaxial layer of SiC devices.

Nowadays, Single-Crystal Diamond (SCD) detectors are used in this field, but their high cost, the small dimensions of the wafers and a low availability of commercial monocrystalline diamonds allow the use of alternative materials such as SiC. In a previous paper [13] a comparison between SCD and SiC detectors was reported, observing an increase in resolution for SCD detectors with increasing thickness. As in SCDs, fast neutron detection in SiC is based on the collection of e–h pairs produced by charged particles produced by neutron interaction with silicon and carbon nuclei. The interaction scheme is shown in Figure 1. Usually, elastic neutron scattering is possible with all energetic neutrons and, depending on the neutron energy and the reaction energy threshold, various reactions can occur [14]. As described in the literature, these reactions allow the production of ionizing particles in or near the active volume of the detector. These particles contain some of the kinetic information of incoming neutrons, allowing for the detection of neutrons.

The reactions of our interest are typically triggered by the interaction of 14 MeV fast-neutrons, produced by a deuterium-tritium neutron generator, with the Si and C nuclei, including only the most common fast-neutron reactions in SiC. Other more complex reactions will also occur, which result in the emission of two or more particles.

From the analysis of the values of fluence as a function of different thickness, calculated with a FLUKA tool [15], we have observed an increment of fluence when the thickness increase. This phenomenon is connected with the macroscopic cross section (Σ [cm^−1^]) that describes the probability of interaction per unit distance and it is related to the number of reactions occurring in a given time interval. Σ is the inverse of the mean free path (λ [cm]) that is the average distance travelled by a particle in a material before an interaction and both Σ and λ depend on the material and on the particle type and energy as described in the literature [16]. From this set of parameters it is possible to obtain the fluence measured in particles per cm^2^. As a result, the volume of the detector is fundamental to obtain a good yield for the detectors. For this reason, we wanted to get a thicker and larger detector trying to increase the epitaxial thickness and the detector area. As mentioned before, a 100 µm thick 4H-SiC detector was compared with SCD detectors, and its resolution was worse than a thicker SCD (150–500 µm). Thus, our aim is to grow a good quality thick epi-layer in order to increase the sensitivity of 4H-SiC detectors. Furthermore, to collect all the charges generated in this thick epitaxy, it is necessary to have a high diffusion length of the same order or larger than the thickness of the epitaxial layer.

In the present work, a 250 µm epitaxial layer of 4H-SiC material was grown through a CVD process and a Chemical Mechanical Polishing (CMP) process at the end of growth. Optical characterization through Photoluminescence (PL) and Raman (i-LOPC) spectroscopy was done for defect distribution and carrier lifetime [17] evaluation. We compared the 250 µm thick epitaxial layer with two 100 µm epi-layers obtained with two different growth rates, 60 and 90 µm/h respectively, on the same reactor (LPE PE106), with the same precursors, the same growth temperatures and a similar quality of the substrates. We also compared the influence of different types of SF defects on the carrier lifetime values. Analysis of the thick epitaxial layer is needed to understand whether this epitaxial material could be used in order to fabricate devices for neutron detection.

## 2. Materials and Methods

### 2.1. Experimental Setup

A HR800 integrated system by Horiba Jobin Yvon in a back-scattering configuration was used for the acquisition of Micro-Raman and PL maps. A He-Cd laser with a wavelength of 325 nm, and with power ranging from 0.15 to 15 mW was used as the excitation source. The power density ranging from about 0.023 to 23.6 kW/cm^2^, respectively. The laser was focused on the sample through an x40 objective and then reflected on a kinematic grating with 1800 grooves/mm. The accuracy of the instrument at room temperature (±1 °C) is about ±0.2 cm^−1^. The spatial resolution achievable with the motorized stage is 0.5 µm, also used for spot laser definition. The laser spot diameter is about 9 µm and this measure was obtained realizing a metal line on the 4H-SiC surface and detecting the reduction of the Raman signal during the line mapping orthogonal to the metal line. Each spectrum was collected with an acquisition time of about 12 s, so to obtain an appreciable signal. For the lowest used laser power (0.15 mW), we have increased the acquisition time (20 s) in order to obtain a good signal intensity.

Deifferent epi-layer thicknesses of 4H-SiC (from 100 to 250 µm) with doping concentrations below the Raman sensitivity limit (*n* < 10^17^ at/cm^−3^ [18]) grown on 4° off-axis Si-face substrates were analyzed.

### 2.2. Epitaxial Growth

The hot-wall reactor (PE106 built by LPE Epitaxial Technology) was used for epitaxial layer growth. The chamber was designed to reduce the temperature ramp-up and ramp-down time and particle formation. The substrates were 4H-SiC (0001), Si face, *n*-type (≅10^18^ cm^−3^) and off-axis (≅4° off towards the [11,12,13,14,15,16,17,18,19,20] direction). These wafers were loaded into the reactor through a load lock system that reduced the background doping level to a value of about 10^13^ cm^−3^ and the growth began with a hydrogen etch sequence. At the deposition temperature the growth precursors are introduced into the hydrogen carrier gas flow (100 slm) and the growth starts at a fixed pressure of 100 mbar. Trichlorosilane (TCS) was used as the silicon source, Ethylene as the carbon source and nitrogen or trimethylaluminum (TMA), respectively, for n-type and p-type doping. More details of this process can be found in reference [19]. The growth rate for the 250 µm thick sample was fixed at 60 µm/h, while it was varied between 60 and 90 μm/h for the 100 μm thick epitaxies.

## 3. Results and Discussion

**Figure 2:**
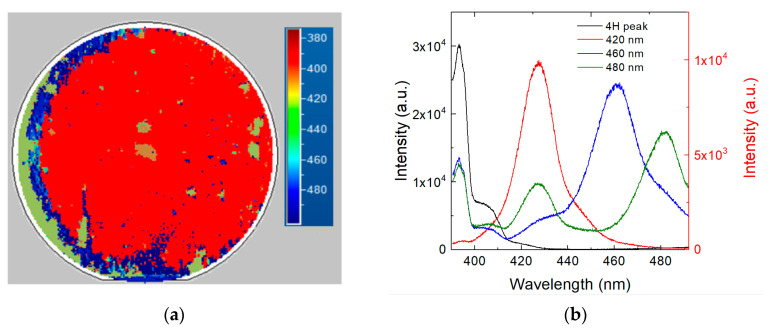
Photoluminescence map (**a**) and relative stacking fault peaks (**b**) of 4H-SiC with epitaxial layer thickness of 250 µm. The black peak represents the band-to-band energy while other peaks are different kinds of SFs, located on the left edge of the wafer. The y-scale on the left is relative to the 4H-SiC region without SFs, while the y-scale on the right is relative to the different SFs.

The optical characterization on the 250 µm thick epi-layer was performed by room-temperature photoluminescence spectroscopy. As shown in Figure 2, most of the defects are located on the left edge of the wafer (2a), highlighted by blue and green colours. This effect is connected to the presence of the off-axis of the substrate towards the [11,12,13,14,15,16,17,18,19,20] directions. With this off-axis the left side of the wafer does not present a lateral growth of the steps and more defects are generated in this region. This behaviour is more evident in the thick epitaxial layers and less evident in the thin ones. The red portion of Figure 2 represents the SF-free zone as it shows only the typical 4H-SiC band-to-band peak (3.23 eV).

Apart from the band-to-band peak at about 390 nm, the other three peaks related to defects can be observed, alongside different kinds of in-growth stacking faults, at 430 nm, 460 nm, 490 nm. The staking sequence was determined as the (4,4) and (5,3) types in the Zhdanov’s notation for 460 nm and 490 nm peaks respectively [20]. The peak at about 430 nm could be related to a bar-shaped staking fault or a single Shockley stacking fault because both exhibit a similar emission wavelength [21]. Some of these stacking faults (SF) are generated from the substrate due to the basal plane dislocations (BPD) but they can also be produced in the epi-layer during the growth, for example due to the presence of impurity deposited on the sample. This kind of defect can affect the reliability and the performance of semiconductor devices owing to the increase of leakage current and the reduction of the carrier lifetime. Therefore, the red zone of the wafer in Figure 2 is the area with the lowest number of defects and represents the useful part for the realization of the devices.

One of the main parameters of the material that strongly affects the performance of the detector is the carrier lifetime. Hence, to collect all the charges generated by neutrons, it is necessary that these charges can reach the contacts and then a high diffusion length is needed in these very thick detectors. To obtain a good measurement of this parameter, several techniques can be used, starting with the micro Photo Current Decay (µPCD), or the time resolved photo luminescence (TRPL) [22]. An alternative method to define the carrier lifetime is Raman spectroscopy, in particular the Longitudinal Optical Phonon-Plasmon Coupling mode (LOPC) [17]. The LO phonon mode, due to the atomic displacement associated with it, allows the interaction between nearby electrons generating a longitudinal electric field which, due to high intensity of free carriers, leads to an additional interaction between the free carriers and the LO phonon mode [23].

**Figure 3:**
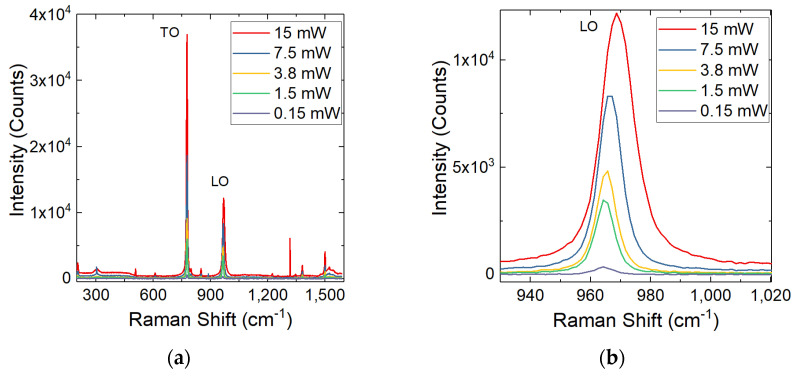
Raman spectra for 250 µm sample analyzed at different laser powers (**a**) and a focus on LO peak (**b**). The LO shift can be observed while TO peak is always in the same position.

As a consequence, there can be observed an LO shift, while the transverse optical (TO) peak always maintains the same position, as shown in Figure 3. The following measurements were taken away from defects, in relatively clean areas. These results will be compared with the values obtained in the vicinity and on the various types of isolated SFs.

This interaction is widely described in the literature [24,25,26] through a mathematical model that allows us to determine the doping [18,27] and the electron mobility [28] of thin films. Micro-Raman analysis was also used in conjunction with the Hall effect and transmission measurements to extract carrier density profiles from SiC wafers [17].

This Raman methodology analysis, called induced-LOPC (i-LOPC), was used in the present work to study the epitaxy quality. The electron plasma, generated by the source, interacts with the vibrations of the lattice by inelastic scattering and this interaction is strongly correlated to the plasmon frequency (1) and the plasmon damping constant (2), defined by the following equations:

ω_p_^2^ = 4πηe^2^/e_inf_m* (1)

γ = e/(m*µ)(2)

where m* is the electron effective mass, e is electron charge, e_inf_ is the high frequency dielectric constant and µ is the carrier mobility [25,26].

From these equations, it is possible determine the residual carrier density and carrier mobility thanks to the LOPC frequency shift and FWHM obtained by Raman spectra fitting. However, due to carrier concentration dependence of the plasmon damping constant, the Raman shift of the LOPC mode, in cases of low doped samples (*n* < 10^18^ cm^−3^), provides a reliable electron concentration, where the LOPC Raman mode is insensitive to the changes in the plasmon damping constant.

It is important to note that, as described in the literature, the number of free carriers depends on the generation (G) and recombination (U) conditions of the electron-hole (e-h) pairs. In the thermodynamic equilibrium condition, G and U are equivalent and therefore this condition can be described as follows:

G = U = η − η_0_/τ (3)

where η_0_ and η are the initial carrier concentrations and the equilibrium carrier concentrations induced by laser respectively, τ is the minority carrier lifetime and G is the injection of carriers generated by laser exposure. Furthermore, the generation of carriers, in our case, depends on various factors related to both the material and the laser parameters, as described by the following expression:

G = φαe^αx^ (4)

where φ is the flux applied by the laser, α is the absorption coefficient, and x is the distance within the epitaxy at which the carriers are generated. From this set of equations, under certain conditions, it is possible to extract the carrier lifetime. It is important to note that this method is useful in the case of high carrier injections (φ > 10^17^) and for low-doped epitaxial layers, about 10^15^ cm^−3^, otherwise it is not possible to observe substantial carrier generation.

The Raman shift at different laser powers was observed. The LOPC mode was acquired for a low-doped epitaxial layer with 250 µm thickness (η about 10^14^ at/cm^3^) 4H-SiC film at different laser powers (from 0.15 to 15 mW).

**Figure 4:**
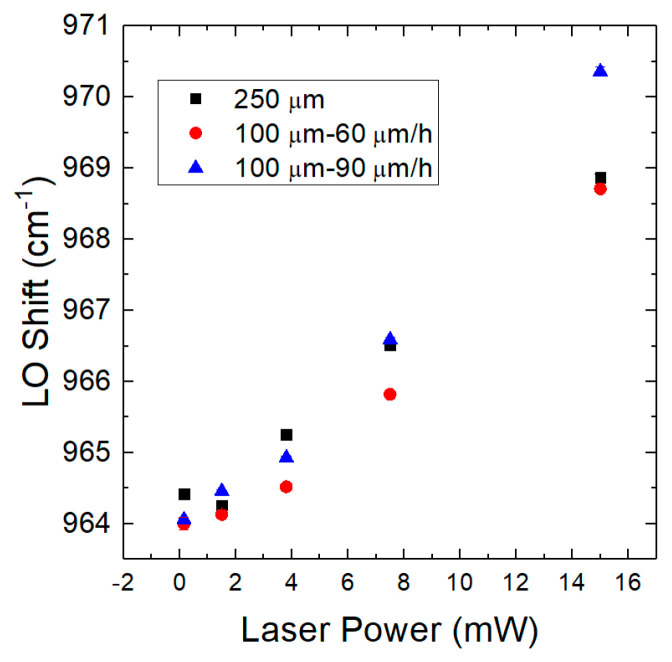
Plot of the Raman shift as a function of laser power.

As can be seen in Figure 4, by increasing the laser power, an increase in the Raman shift (LO peak) is obtained and consequently there is also an increase in the laser-induced carrier density. The LO peak underwent a significant shift towards high frequencies due to the increase in e-h pairs generated as the laser power increases. Through the analysis of the LOPC, the free carrier concentration was obtained [25,26,29], and Table 1 shows the results.

As shown in the Table 1, diffusion length values were calculated, however these values are much lower than the epitaxial thickness, necessary for a good detection yield of the detector. There are some processes to increment the carrier lifetime and the diffusion length for 4H-SiC. A post thermal growth process, such as oxidation and passivation, could increase these values thanks to the decrement of carbon vacancies [30].

A subsequent analysis was conducted in order to compare the above data for 250 µm thick layer with other 4H-SiC samples having different thickness or different growth rate. A determination of carrier lifetime and residual carrier density was done for other two samples with an epitaxial layer of 100 µm. Table 2 shows carrier lifetime results for all samples. These 100 µm samples were previously grown epitaxially with the same CVD process in the same horizontal hot-wall reactor with a similar process.

As a result, the carrier lifetime decreases with the increment of laser power. The two samples with the same thickness (100 μm) and different growth rates show a better carrier lifetime in the case of the lower growth rate (60 μm/h). This behaviour is related to a higher concentration of point defects at a higher growth rate that has been previously predicted by Monte Carlo simulations [31,32] and then confirmed by experimental results [33]. The decay of the quality of the epitaxial growth with increasing thickness has been observed in a previous work [34] where a decrease in the yield of the Schottky diodes was observed for the same epitaxial process, increasing the thickness from 30 to 80 μm, due to the increase of the leakage current of the diodes. In this work we have observed instead a deterioration of the carrier lifetime due to the increase of point defects, probably related to the roughness increase at the surface of the epi-layer due to the step bunching observed in 4H-SiC.

Moreover, for an injection level under 10^18^ cm^−3^, the radiative contribution can be neglected, so that the Auger recombination (AR) can be neglected too [22]. When the concentration of induced carriers increases, the Auger recombination mechanism becomes more and more important, passing first through a monomolecular recombination mechanism and then through a bimolecular one, as the carrier concentration increases [35]. Probably, due to the high laser powers used, a greater concentration of carriers is induced, and a shorter lifetime due to recombination mechanisms is obtained.

As described in the literature [36], for the highest concentrations, in a high injection regime, the bimolecular recombination rate is presumably contributed by both band-to-band radiative and trap-assisted Auger recombination mechanisms, where the carrier lifetime is lower. It is important to note that surface recombination and point defect recombination contributions are not distinguished by this method and also the temperature could affect diffusivity and recombination rates [37].

**Figure 5:**
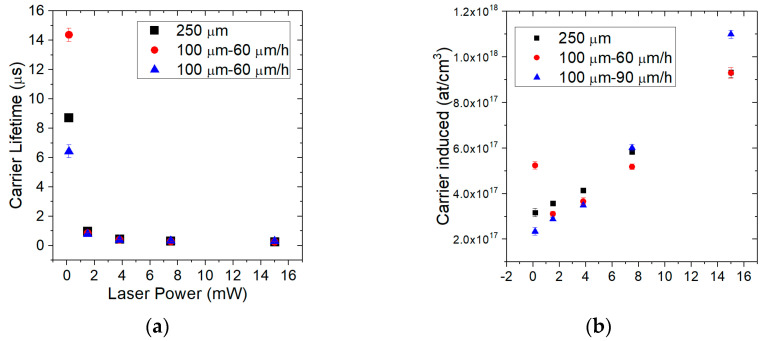
The figure shows the trends of the carrier lifetimes (**a**) and the amount of carrier induced (**b**) as a function of Raman shift and laser power for all samples analysed.

In the Figure 5, the trends of the carrier lifetime for all the analysed samples are shown.

As the values listed in Table 3, in this figure the carrier lifetime increment when the laser power decreases is reported.

As stated above, the carrier lifetime analysis was done in SF-free areas. In order to estimate how different types of SFs can affect the increase of free carriers and the carrier lifetime trend, several PL and Raman maps were acquired on SFs in order to compare them with data acquired far from defects. As shown before (Figure 2b), we have different kinds of SF defects that can influence in different ways the characteristics of 4H-SiC devices. Thanks to photoluminescence spectroscopy, it is possible to detect and analyse different crystalline defects inside the epi-layer, which act as recombination centres and, in some cases, we could define whether the SF defects start from the interface, in the basal planes. In general, considering the width of the bars, for the bar shaped SFs, we could calculate the depth of the defect, knowing the off angle and epitaxial thickness [21].

**Figure 6:**
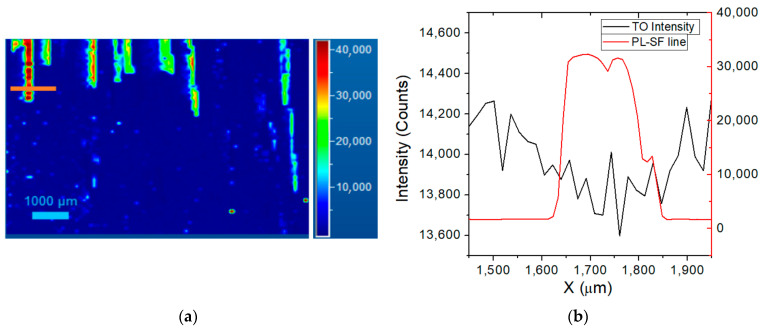
PL map of an isolated defective zone (SF at 430 nm) (**a**), and the result of the Raman map line compared with the TO intensity variation (**b**), using a laser power of 15 mW.

Figure 6 shows a PL map for a SF with energy of 2.88 eV (430 nm) and a variation of TO intensity on and out of SF using high laser power.

**Figure 7:**
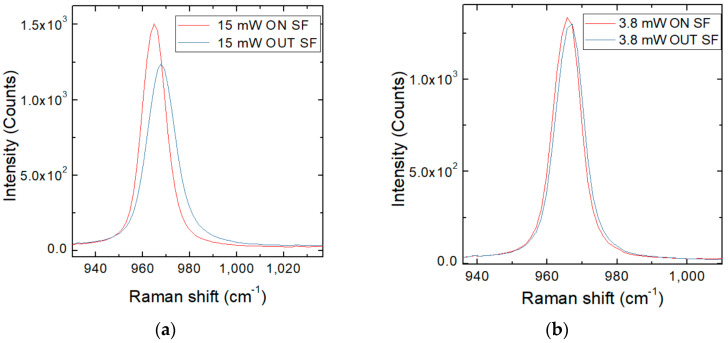
The figure shows LO Raman Shift on, and in proximity of, isolated SF defect at 430 nm (2.88 eV) with a laser power of 15 mW (**a**) and 3.8 mW (**b**) observing a reduction on shift.

A Raman line across the isolated defect was done, as defined in Figure 6a, using different laser powers in order to determine the carrier density and the carrier lifetime on the SF and in its proximity. It was performed as the same analysis on different SFs defects, with different energies, 2.43 (510 nm) and 2.53 eV (490 nm), respectively. It was observed that using high laser power (15 mW), the spectrum acquired on the SF showed a strong Raman shift reduction, suggesting a trapping of free carriers. As shown in Figure 7, for the lower laser powers used, we observed that under 3.8 mW, there are no differences between the ON SF and OUT SF spectra.

**Figure 8:**
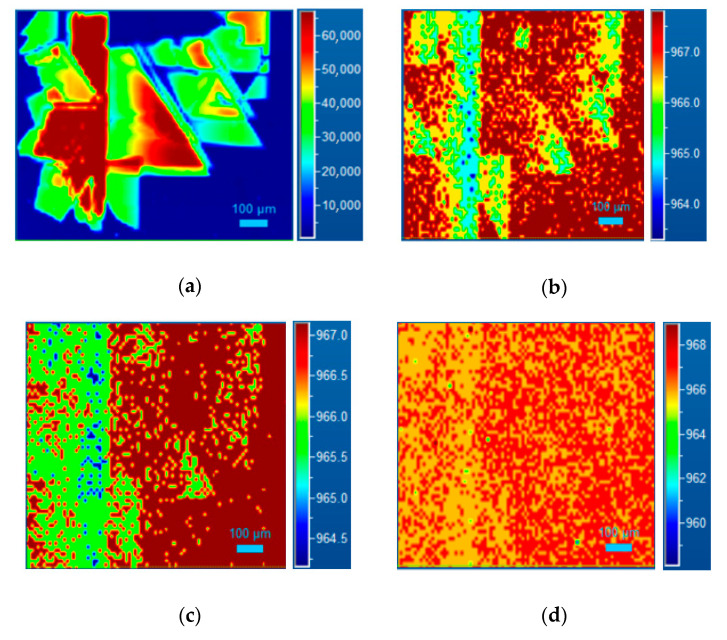
PL map (**a**) and Raman map following LO peak shift for a SF at 2.88 eV (430 nm), at different laser powers: 15 (**b**), 7.5 (**c**) and 3.8 mW (**d**) respectively.

This suggests that the same values of carrier induced, and the lifetime in and out, of the defect (in proximity) are obtained when low laser power is used. This difference is more evident by the Raman maps, following the LO peak shift as shown in Figure 8.

As the laser power decreases, a reduction of the LO Raman shift is observed. This effect is probably due to the fact that if the carrier lifetime and the diffusion length increases and then this measure is mediated in a larger region around the SFs, while the effect of the carrier lifetime and the induced carrier is lower.

Table 3 shows the lifetime values for different kinds of SFs and for different laser powers compared with the defect-free areas far from the stacking faults.

As shown in the table above, carrier lifetime data for all SFs are lower than in the clean area.

The most common long-range defect in our sample is the SFs at 430 nm in the PL map, the other two, 490 and 510 nm, are generally located on the edge of the wafer, as shown in Figure 2a.

From these results it is clear that Raman spectroscopy is a powerful tool for careful characterization of 4H-SiC epitaxial layers. By exploiting the i-LOPC method it is therefore possible to define parameters such as doping, mobility and lifetime of carriers in the epitaxial layers. Moreover, the different types of defects act in a slightly different way on the carrier lifetime, leading to a recombination or trapping of the free carriers.

## 4. Conclusions

The epitaxial growth of a 4H-SiC wafer with a thickness of 250 µm and a deep characterization to evaluate the quality of the epi-layer was performed. For this purpose, we have used room-temperature PL spectroscopy and the micro-Raman spectroscopy by using the i-LOPC method, a non-destructive technique to determine the general quality of epitaxy.

For the carrier lifetime values, different behaviours on different samples depending both on growth rate and thickness were obtained. The induced carrier lifetime is increasing with the decrement of laser power and also by lowering the carrier density according to the theory and other experimental data.

From our measurements it is clear that the presence of SFs in the material has a large effect at high carrier injection but at low carrier injection it is negligible. The 250 µm sample has a low percentage of SFs defects, but they are located on the left edge of the wafer due to the off-axis angle and epitaxial growth conditions. The low impact of the SFs on the carrier lifetime at low carrier injections is extremely important for the detector applications, where the carrier injection due to the particles is extremely low. Finally, the developed process of the 250 µm epitaxial layer is useful for improving the performance of previous devices studied with lower epitaxial thicknesses (100 µm) [13], as an increase in the thickness, and therefore in the volume of the detector, leads to an increase in responses for neutron detection. Some additional processes like high temperature oxidation and annealing [30] should be implemented to further increase the diffusion length of this type of material.

## Figures and Tables

**Table 1:** LO Raman Shift, carrier density, carrier lifetime and diffusion length as a function of laser power.

Laser Power (mW)	Peak Center (cm^−1^)	Carrier Density, *n* (At/cm^3^)	Carrier Lifetime (μs)	Diffusion Length (μm)
0.15	964.4	3.17 × 10^17^	8.71	47.88
1.5	964.3	3.57 × 10^17^	0.98	16.02
3.8	965.2	4.15 × 10^17^	0.45	10.54
7.5	966.5	5.83 × 10^17^	0.32	8.43
15	968.8	9.32 × 10^17^	0.26	6.75

**Table 2:** Carrier lifetime data for each sample as a function of laser power.

Laser Power (mW)	τ (µs) 100 μm 60 µm/h	τ (µs) 100 μm 90 µm/h	τ (µs) 250 µm 60 µm/h
0.15	14.38	6.43	8.71
1.5	0.85	0.80	0.98
3.8	0.40	0.38	0.45
7.5	0.28	0.33	0.32
15	0.26	0.30	0.26

**Table 3:** Carrier lifetime data on different SFs defect compared with values on the clean area.

Laser Power (mW)	τ (µs) 430 nm Peak	τ (µs) 490 nm Peak	τ (µs) 510 nm Peak	τ (µs) Clean Area
0.15	5.41	3.46	4.43	8.71
1.5	0.68	0.49	0.48	0.98
3.8	0.31	0.23	0.24	0.45
7.5	0.23	0.17	0.17	0.32
15	0.18	0.14	0.15	0.26

## Data Availability

The data presented in this study are available on request from the corresponding author.
